# Head and Neck Malignancies in Autoimmune Polyendocrine Syndrome Type 1 (APS-1/APECED): A Scoping Review of Molecular Pathogenesis, Clinical Features, and Outcomes

**DOI:** 10.3390/ijms26188969

**Published:** 2025-09-15

**Authors:** Marko Tarle, Marina Raguž, Ivica Lukšić

**Affiliations:** 1 Department of Maxillofacial Surgery, Dubrava University Hospital, 10000 Zagreb, Croatia; tarlemarko1@gmail.com; 2 School of Dental Medicine, University of Zagreb, Gundulićeva 5, 10000 Zagreb, Croatia; 3 Department of Neurosurgery, Dubrava University Hospital, 10000 Zagreb, Croatia; marinaraguz@gmail.com; 4 School of Medicine, Catholic University of Croatia, 10000 Zagreb, Croatia; 5 School of Medicine, University of Zagreb, Šalata 3, 10000 Zagreb, Croatia

**Keywords:** autoimmune polyendocrine syndrome type 1 (APS-1), chronic mucocutaneous candidiasis, head and neck cancer, squamous cell carcinoma

## Abstract

Autoimmune polyendocrine syndrome type 1 (APS-1, APECED) is a rare monogenic disorder caused by biallelic AIRE mutations and is classically associated with chronic mucocutaneous candidiasis (CMC), hypoparathyroidism, and adrenal insufficiency. Apart from the autoimmune manifestations, APS-1 is associated with an increased risk of squamous cell carcinoma (SCC), particularly in the oral cavity and esophagus. However, the evidence is patchy and has not yet been systematically reviewed. We conducted a scoping review according to the PRISMA-ScR guidelines. Pub-Med, Scopus, and Web of Science were searched using the terms APS-1/APECED and malignancy until July 2025. Eligible studies reported on APS-1 patients with histologically confirmed head, neck or esophageal cancer. Clinical, pathological, genetic and outcome data were summarized narratively. Nine publications described 19 APS-1 patients with 26 tumors. The mean age at cancer diagnosis was 35 years, with a latency period of ~24 years from the onset of APS-1. Tumors occurred most frequently in the oral cavity (65%), followed by the lip (19%) and esophagus (15%). In 96% of cases, the tumors were SCC. The grade of the tumor varied, and almost half of the cases were diagnosed at an advanced stage. As far as reported, the usual risk factors were not particularly pronounced; many patients did not smoke or drink alcohol. The main treatment consisted of surgery, often in combination with radiotherapy or chemoradiotherapy, alongside long-term antifungal therapy. Despite the multimodal treatment, outcomes were poor: the overall survival rate was ~50%, with recurrence occurring in 38% of cases and a second primary tumor in 26%. A further 14 cases were reported from another Italian cohort, which together with the national cohort dana suggest a risk of approximately ~10% with APS-1; however, the true lifetime risk remains uncertain. Head and neck malignancies in APS-1 occur early, often without classic risk factors, and have a high recurrence and mortality rate. Lifelong surveillance, antifungal stewardship and increased clinical awareness, ideally as part of multidisciplinary treatment pathways, are critical to improving outcomes in this rare but high-risk population.

## 1. Background

Autoimmune polyendocrine syndrome type 1 (APS-1), also known as autoimmune polyendocrinopathy–candidiasis–ectodermal dystrophy (APECED), is a rare monogenic disorder caused by biallelic mutations in the autoimmune regulator (AIRE) gene on chromosome 21q22.3 [1,2]. The AIRE protein is essential for central immune tolerance, as it drives the ectopic expression of tissue-restricted antigens in medullary thymic epithelial cells, thereby enabling the clonal deletion of autoreactive T cells and supporting the selection of regulatory T cells [3]. Loss-of-function mutations in AIRE disrupt this process, enabling the persistence of autoreactive lymphocytes and promoting widespread autoimmunity with a characteristic autoantibody profile [3,4]. Clinically, APS-1 is defined by the classic triad of chronic mucocutaneous candidiasis (CMC), hypoparathyroidism, and primary adrenal insufficiency, but the phenotypic spectrum extends to a variety of additional endocrine and non-endocrine manifestations, including alopecia, vitiligo, keratitis, enamel hypoplasia, autoimmune hepatitis, hypothyroidism, and premature ovarian failure [1,5]. APS-1 is extremely rare, with an estimated prevalence of ~1 in 2–3 million in the general population. Due to founder effects, much higher rates are observed in genetically isolated populations: approximately 1 in 25,000 in Finland, 1 in 14,000–25,000 in Sardinia, and 1 in 9000 among Iranian Jews [5,6]. The disease typically manifests in childhood, often with CMC as the first clinical sign, and progresses with additional endocrine deficits and autoimmune phenomena in adolescence or early adulthood [7,8]. Improved molecular diagnostics and supportive therapy have increased survival, but APS-1 is still associated with significant morbidity and early mortality, not only from endocrine crises and infections, but also from malignancies [9]. In recent years, there has been increasing evidence that APS-1 patients have an increased risk of squamous cell carcinoma (SCC), particularly in the oral cavity and esophagus. In contrast to sporadic SCC in the head and neck, which typically occur after decades of tobacco and alcohol exposure, APS-1-associated cancers have been reported in younger patients, who often lack these conventional risk factors. This unusual susceptibility to cancer appears to be closely related to the immunological and microbial consequences of AIRE deficiency [10,11]. Neutralizing autoantibodies against IL-17A, IL-17F, and IL-22, which are present in the vast majority of APS-1 patients, significantly impair the Th17 pathway, which is crucial for mucosal fungal defense. As a result, ~90% of patients develop CMC, often in early childhood [12]. Persistent colonization with Candida albicans creates a microenvironment that is not only infectious but also potentially carcinogenic. Mechanistic studies have shown that *C. albicans* can metabolize glucose and ethanol to acetaldehyde. Acetaldehyde is a group I carcinogen in humans, causing DNA adducts and chromosomal instability, even in the absence of exogenous alcohol. Candida can also produce nitrosamines in the oral mucosa, which activate proto-oncogenes and contribute to dysplasia [13]. More recently, the fungal peptide toxin candidalysin, secreted during hyphal growth, has been identified as an important virulence factor that directly violates epithelial barriers and simultaneously activates the EGFR–MAPK signaling axis, promoting proliferation and chronic inflammatory responses. Taken together, these mechanisms converge with canonical pathways of squamous cell carcinogenesis, including TP53 inactivation, CDKN2A/p16^INK4a^ loss, and EGFR-driven signaling, providing a plausible link between AIRE deficiency, impaired immune surveillance, and malignant transformation [14]. In addition to infection, the broader autoimmune milieu of APS-1 may also contribute to carcinogenesis. High-titer autoantibodies against type I interferons (IFN-α/ω) are nearly universal in APS-1 and can impair innate antitumor immunity, allowing nascent neoplastic clones to escape immune surveillance. In addition, chronic autoimmune inflammation in various organs can promote a systemic pro-carcinogenic state via inflammatory mediators such as TNF-α and IL-6. APS-1 may represent a complex immune–infection–cancer axis, in which genetic autoimmunity and persistent fungal colonization potentially interact to contribute to early-onset epithelial cancer [15,16].

Although awareness of these associations has grown, important challenges remain in understanding the clinical impact of malignancies in APS-1. The condition is extremely rare, and most available evidence is limited to isolated case reports or small case series. There is a lack of standardized surveillance strategies, treatment guidelines, and population-based outcome data. These limitations hinder efforts to estimate the true cancer risk, identify modifying factors, or guide clinical decision-making in APS-1 patients.

Despite these findings, knowledge of head and neck malignancies in APS-1 remains fragmented. Most reports are single cases or small series, with limited synthesis between populations. National cohorts from Finland and Italy have indicated an increased risk of cancer, but detailed clinical, pathological, and outcome data are sparse [17,18]. This emphasizes the need to consolidate the published cases to better define clinical features, risk factors, treatment strategies, and prognosis. This scoping review aims to comprehensively map and synthesize published cases of head and neck malignancies in APS-1, describe their demographic, clinical, and pathological features, and explore possible associations with risk factors, treatment strategies, and outcomes to inform future research and clinical awareness.

## 2. Results

### 2.1. Study Selection and Characteristics of Included Studies

The systematic search across PubMed/MEDLINE, Scopus, and Web of Science retrieved a total of 461 records. After removing 65 duplicates, 396 unique records remained for title and abstract screening. Of these, 367 articles were excluded as irrelevant, most commonly because they described non-APS-1 polyendocrine syndromes, malignancies outside the head and neck region, or narrative reviews without primary patient data. A total of 29 articles were retrieved for full-text analysis. Twenty articles were excluded at this stage: seven did not involve APS-1 patients, seven represented inappropriate study designs without case-level information, one described a malignancy outside the head and neck region, and five provided insufficient diagnostic or clinical data. Consequently, nine publications met all eligibility criteria and were included in the qualitative synthesis. They included 19 unique APS-1 patients with histologically confirmed malignancies in the head and neck region. Independently, the Italian national APS-1 cohort reported on 14 additional patients with head and neck malignancies (7 oral mucosal, 3 tongue, 3 esophageal and 1 lip carcinoma) [17]. As no information was available at the individual level, these cases were summarized descriptively and not included in pooled analyses. A total of 33 APS-1 patients with malignant tumors of the head and neck have been documented in the literature to date.

**Figure 1:**
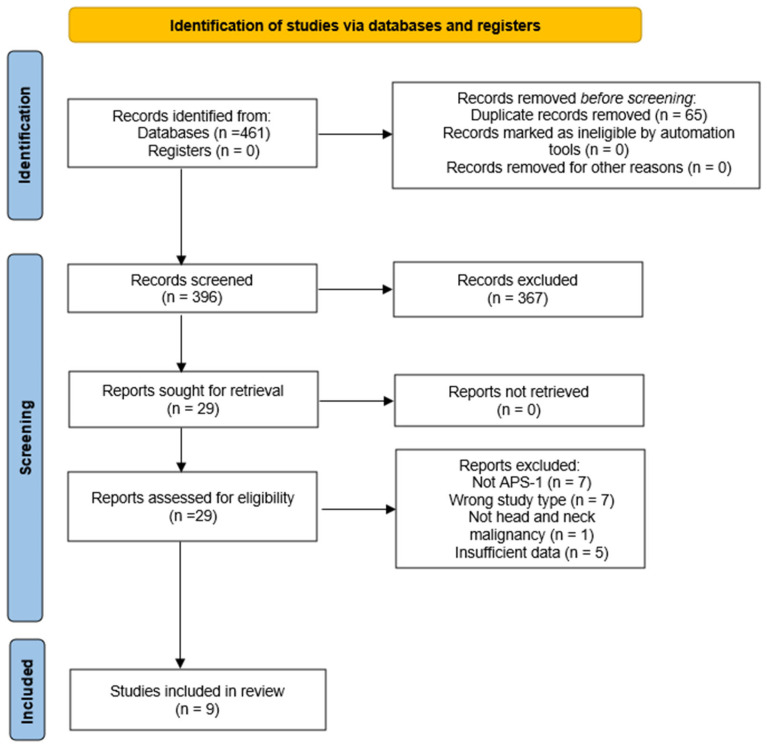
PRISMA-ScR flow diagram of study selection. Flow chart illustrates the number of records identified, screened, assessed for eligibility, and included in the final synthesis.

The process of study identification and screening followed the PRISMA-ScR guidelines, and the detailed flow of records is shown in the PRISMA flowchart (Figure 1) [19].

### 2.2. Demographic and Clinical Characteristics

Among the 19 APS-1 patients included in the pooled analysis, 11 were male (57.9%) and 8 were female (42.1%), resulting in a slight male preponderance. The median age at cancer diagnosis was 35 years (mean 35.7, range 21–58), which was significantly younger than the average age at diagnosis of sporadic squamous cell carcinoma of the head and neck. In 12 patients with available information on the occurrence of APS-1, the median age of first disease manifestation was 10.5 years, and the median interval between the occurrence of APS-1 and cancer diagnosis was 24 years. This illustrates the typically long latency period between the onset of autoimmunity and malignant transformation. The cases were predominantly from Northern Europe (Finland, n = 8; Norway, n = 4), with additional reports from Canada (n = 1), USA (n = 1), Israel (n = 1), Ireland (n = 1), Austria (n = 1) and Australia (n = 2) [9,20,21,22,23,24,25,26,27]. This distribution reflects both the high prevalence of APS-1 in founder populations and sporadic cases worldwide.

### 2.3. Clinical Features of APS-1

Regarding the classic triad of APS-1, chronic mucocutaneous candidiasis (CMC) was found in 17 patients (89.5%), hypoparathyroidism (HP) in 16 patients (84.2%) and primary adrenal insufficiency (PAI) in 17 patients (89.5%). Several additional autoimmune or ectodermal manifestations were also noted, including alopecia (A) (57.9%), enamel hypoplasia (EH) (26.3%), vitiligo (V) (26.3%), keratitis (K) (21.1%) and premature ovarian failure (POF) (21.1%). Less common features included diabetes mellitus (DM), hypothyroidism (HT) and pernicious anemia (PA). Taken together, these findings suggest that the majority of patients had developed multiple components of APS-1 beyond the defining triad at the time of cancer diagnosis. When reported, CMC long predated cancer: the median documented CMC duration was 24 years (IQR 15.9) in 16 patients with data.

### 2.4. Genetics and Autoantibodies

Genetic confirmation of APS-1 was present in nine patients (47%), while the others were diagnosed on clinical grounds. The most common pathogenic variant was the c.967_979del13 deletion, which was identified in four individuals (three from Norway and one from Austria), all in homozygous form [9,23]. The second most common was the R257X mutation, which was found in three Finnish patients, also in homozygous form [22,27]. In addition, one Israeli patient carried the rare A374G/A374G variant [27], and another Norwegian patient had a composite genotype with a combination of c.22C>T and c.402delC [9]. Autoantibody tests were available for the same nine patients with genetic confirmation. All had characteristic interferon-ω autoantibodies, while most also had broader autoreactivity, including IL-22, 17α-hydroxylase, 21-hydroxylase, aromatic L-amino acid decarboxylase (AADC), tryptophan hydroxylase 1 (TPH1), SOX10, NALP5 and squamous cell carcinoma (SCC) antibodies. These findings are consistent with the well-established serological profile of APS-1, which is characterized by a broad spectrum of autoantibodies against cytokines and steroidogenic enzymes.

### 2.5. Tumor Location, Grade and Stage

A total of 26 malignant tumors were identified in 19 APS-1 patients, reflecting the occurrence of multiple primary tumors in several individuals. The oral cavity was the most common site (17/26 tumors, 65%), including the tongue (6 tumors), buccal mucosa (7), gingiva (3) and hard palate (1). In addition, 5 tumors (19%) originated from the lower lip, while 4/26 tumors (15%) were esophageal carcinomas. Histology revealed squamous cell carcinoma in 25/26 tumors (96%), while 1 tumor (4%) was an adenocarcinoma of the esophagus, which was reported as poorly differentiated. Tumor grade was available for 17 tumors: 9 were well differentiated (grade 1, 53%), 4 were moderately differentiated (grade 2, 24%) and 4 were poorly differentiated (grade 3, 24%). Most tumors were well differentiated. Stage information was available for 15 tumors: 4 were stage I, 3 were stage II, 3 were stage III and 5 were stage IV. Accordingly, 7 tumors (47%) were diagnosed at an early stage (I–II), while 8 (53%) had advanced disease (III–IV).

### 2.6. Conventional Head and Neck Cancer Risk Factors

The majority of patients had information on the usual risk factors. A smoking history was obtained in 14 individuals: 5/14 (36%) were smokers, of whom 2 were heavy smokers (≥20 cigarettes per day over many years), 1 was a light smoker and 2 were occasional or minimal smokers. The remaining 9/14 (64%) had never smoked. Alcohol consumption was recorded in the same subgroup. 8/14 patients (57%) consumed alcohol, with 3 categorized as heavy drinkers (regular, high consumption) and 5 as minimal or occasional drinkers. The remaining 6/14 (42%) did not consume alcohol. When smoking and alcohol were considered together, only a minority of patients had both established risk factors in a clinically relevant range. In contrast, more than half of the cohort (at least 8 patients, ~57%) neither smoked nor consumed alcohol and still developed invasive squamous cell carcinoma of the oral cavity or esophagus. This distribution underscores an important finding: the majority of APS-1 patients with head and neck cancers did not have classic environmental risk factors typically associated with these malignancies. Instead, their cancers developed in the context of long-standing chronic mucocutaneous candidiasis, suggesting that persistent fungal infection and localized inflammation, rather than tobacco or alcohol, are the main carcinogenic factors in this patient population.

### 2.7. Treatment Modalities and Adjunctive Antifungal Therapy

The therapeutic strategies varied depending on the location of the tumor and the stage at diagnosis. Surgery was the primary treatment in 9 of 19 patients (47%). In most cases, this involved hemiglossectomy or partial glossectomy for tongue carcinoma, wide local excision for buccal mucosal tumors, neck dissection for patients at high risk of occult metastases due to locally advanced disease or nodal involvement, and esophagectomy for esophageal carcinoma. Primary radiotherapy was given to 1 patient and primary chemotherapy to 2 patients, while 1 patient received only palliative radiotherapy. In addition, 1 patient received neoadjuvant radiotherapy followed by surgery and another received neoadjuvant chemoradiotherapy prior to surgery. One patient died from the rapidly progressing disease before treatment could be initiated and in 3 cases no information about the treatment was available. Of the surgically treated patients, 3 subsequently received adjuvant radiotherapy or chemoradiotherapy due to advanced disease stage or positive surgical margins. In addition, 9 patients were treated with long-term antifungal therapy in addition to oncological treatment. The active substances included fluconazole, ketoconazole, voriconazole, miconazole, nystatin and amphotericin B. Antifungal therapy was used both to control chronic mucocutaneous candidiasis and to reduce the fungal burden during and after oncological treatment. Supportive and palliative treatment was reported in several patients with disseminated or unresectable disease, including palliative radiotherapy for esophageal cancer and combined antifungal and nutritional support for end-stage oral SCC. Overall, the therapeutic approach was consistent with standard oncological practice for SCC of the head and neck but was consistently accompanied by aggressive antifungal prophylaxis or treatment to detect the underlying APS-1-associated candidiasis.

### 2.8. Outcomes, Follow-Up, Recurrence and Second Primary Tumors

**Figure 2:**
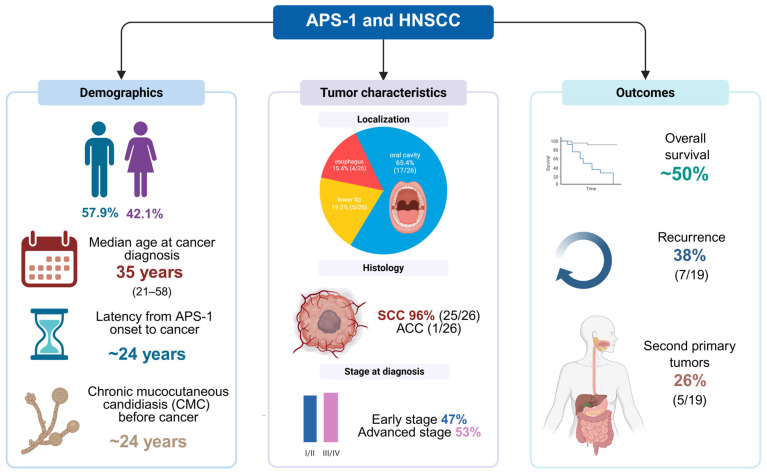
Head and neck malignancies in APS-1: summary of reported cases. Overview of clinical characteristics, tumor features, and outcomes of all APS-1 patients with head and neck cancers described in the literature. ACC—adenocarcinoma; SCC—squamous cell carcinoma; HNSCC—head and neck squamous cell carcinoma; APS-1—Autoimmune Polyendocrine Syndrome type 1. Created with www.app.biorender.com (accessed on 30 August 2025).

The follow-up period in the available reports ranged from 5 to 307 months (median approximately 60 months) and illustrates the marked variability of disease progression in APS-1 patients who developed malignant head and neck tumors. During this period, 9 of 19 patients (47%) died, all because of progressive malignancy, while 10 of 19 (53%) were still alive at last follow-up, although one of these survivors was described as terminal at the time of reporting. Thus, the overall survival rate in this cohort was nearly 50%, reflecting the aggressive natural history of these cancers in APS-1. When the results were stratified by stage at presentation, a clear pattern emerged. Among patients with early-stage disease (stage I–II), only 1 of 6 (17%) died, and most achieved long-term survival. In contrast, among patients with advanced tumors (stage III–IV), 5 out of 8 patients (63%) died during follow-up, highlighting the poor prognosis associated with locoregional spread or nodal involvement. These results show that stage at diagnosis is the most important determinant of outcome, with early detection offering the best chance of long-term survival. The longest follow-up was documented in a Finnish woman with tongue SCC who was alive and disease-free 25 years (307 months) after initial surgical resection. Several other patients survived longer than 5 years, including patients treated for localized tongue and buccal mucosa SCC. In contrast, patients with advanced esophageal cancer or multifocal oral SCC usually died within 1–3 years despite multimodal therapy. Local recurrence was observed in 7 of 19 patients (37.8%), with most recurrences occurring within the first 12–24 months after initial treatment. Recurrences were documented both after surgery alone and after multimodal therapy. Second primary tumors (SPTs) were found in 5 of 19 patients (26.3%). Most of the SPTs were additional SCCs at different oral sites, including lip, gingiva and commissure. In one case, carcinoma in situ (CIS) developed at the lip margin three years after the original buccal SCC. Another patient with an esophageal SCC later developed a rectal adenocarcinoma, which represents an extracranial SPT. To facilitate comparison between the reports, the key demographic, clinical, pathological and outcome data of all included cases are summarized in Figure 2.

### 2.9. Additional Italian Cohort Data

In the largest national series published to date, the Italian cohort included 158 APS-1 patients who were followed up for an average of 23.7 ± 15.1 years (range 1–67). During the follow-up period, 23 patients (14.6%) developed a total of 25 malignancies, and cancer was the main cause of death in one third of the deceased patients. Of these malignancies, 14 were in the head and neck region: 7 oral cavity carcinomas, 3 tongue carcinomas, 3 esophageal carcinomas and 1 lip carcinoma. The histology was most frequently squamous cell carcinoma. The mean age at cancer diagnosis was relatively young, 42 ± 9.6 years (range 24–68). In terms of mortality, the overall mortality rate in the cohort was 14.6%, and cancer was the immediate cause of death in 8 patients (35% of all deaths), including 2 with metastatic SCC of the oral mucosa, 3 with gastric carcinoma, 2 with esophageal carcinoma and 1 with Kaposi’s sarcoma. Thus, malignancy was a major cause of premature death in APS-1, along with fulminant hepatitis and adrenal crisis. The authors conclude that the risk of malignant tumors is increased in APS-1 (≈14% in their cohort) and that oral, lingual and esophageal carcinomas are the most common tumor types. A strong association with long-standing CMC was emphasized, which is consistent with findings from other populations [17].

In summary, head and neck malignancies in APS-1 occurred at a young age (median 35 years), typically after a long latency period of more than two decades since the first autoimmune manifestations. The vast majority of tumors were squamous cell carcinomas, which often occurred without classic risk factors such as smoking and alcohol. Despite treatment, the overall survival rate was around 50%, with a high rate of recurrence (37.8%) and second primary tumors (26.3%), underlining the aggressive nature and lifelong cancer risk in this patient group.

### 2.10. Summary Table

Table 1 and Table 2 consolidates the extracted data from all included cases, detailing demographic variables, APS-1 diagnostic method, CMC duration, tumor site and histology, risk factors, treatment modalities, outcomes, and recurrence status.

## 3. Discussion

This review summarizes the largest group of APS-1-associated malignancies of the head and neck reported to date and highlights several characteristic clinical and pathological patterns. Across 19 unique patients (26 tumors) from nine publications, the median age at cancer diagnosis was 35 years (range 21–58), decades earlier than the typical incidence of sporadic head and neck squamous cell carcinoma (HNSCC), which usually occurs after the sixth decade of life [28,29,30]. In addition to these cases, the Italian national APS-1 cohort reported a further 14 patients with head and neck cancer, bringing the total number of documented APS-1 patients with oral, lip or esophageal cancer to 33 [17]. As individual-level data were not available from the Italian cohort, these cases were not included in our pooled analysis, but they provide important epidemiological context. Importantly, cancer was the most common cause of death in the Italian cohort, accounting for ~35% of all deaths. The cumulative incidence of head and neck malignancies was ~9% of APS-1 patients, similar to the ~10% prevalence in the Finnish registry (6 of 91 patients over 25 years; mean cancer onset 37 years; four cancer-related deaths) [9,17]. These national data, in conjunction with our pooled case analysis, converge to a 10% lifetime risk of head and neck cancer in APS-1, emphasizing that the risk is both reproducible and clinically significant. In our series of 19 patients, we observed a roughly equal distribution between men and women. This contrasts with the clear male predominance in sporadic oral SCC, where men are typically affected almost twice as often as women (ratio 1.5:1 to 3:1 depending on geography and exposure) [31,32]. The gender balance in APS-1-associated cancers suggests that the autoimmune background, which is consistent with the fact that autoimmune disease is significantly more common in women (≈75–80% of cases), may override the male bias in carcinogen-associated SCC [33,34]. This reinforces the interpretation that immune system dysregulation and chronic infections, rather than lifestyle exposure, are the main etiological factors. The tumor localization showed a different pattern. In APS-1, carcinomas were predominantly located in the oral cavity (65%), with sub-points including the tongue, buccal mucosa, gingiva and hard palate, followed by the lip (19%) and esophagus (15%). Remarkably, no patient developed carcinoma of the floor of the mouth, a classic sublocalization in sporadic HNSCC among smokers and heavy drinkers [35,36]. Instead, the tumors clustered at sites typically colonized by Candida albicans in chronic mucocutaneous candidiasis (CMC), supporting the etiological role of persistent fungal infection [37]. The histopathology was predominantly squamous cell carcinoma (96%), with a poorly differentiated adenocarcinoma of the esophagus as the only exception. About half of the tumors were diagnosed at an advanced stage (III-IV), and the tumor grade ranged from well to poorly differentiated. Multifocality was a striking feature. The most dramatic example was described by Richman et al. where a 26-year-old man presented with four concurrent oral SCCs (lip, buccal mucosa, palate and gingiva) [20]. Other patients developed synchronous or metachronous tumors, such as an Austrian woman with simultaneous buccal and lip SCC and several cases of second primary tumors on the gingiva or lip border within a few years [23]. Recurrence occurred in more than one third of patients, often within one to two years after initial treatment. Taken together, these findings suggest the presence of “field cancerization”, but unlike sporadic disease, the field defect here appears to be due to chronic Candida colonization and inadequate immune surveillance [38]. Conventional risk factors were rarely present. More than half of the patients had neither smoked nor drunk alcohol in the past, and among smokers, alcohol consumption was generally low. This is in stark contrast to sporadic oral SCC, where tobacco and alcohol together account for the majority of cases [39]. The high proportion of APS-1 cancers in risk factor-negative individuals (≈57%) emphasizes the independence of this association from classical carcinogens. Instead, the carcinogenic drivers in APS-1 are intrinsic immune dysfunction, chronic mucosal infections and persistent inflammation. The results were poor. Early-stage tumors that were treated surgically could be brought under control, but advanced tumors often progressed despite multimodal therapy. In all cases, about half of the patients died of their cancer, which is consistent with the high mortality in national cohorts [9,17]. Survivors often require repeat interventions for recurrent or metachronous disease. These data confirm that APS-1 patients are not only predisposed to develop early-onset SCC but also have a lifelong risk of multiple cancers and poor long-term survival. The overall picture is consistent: APS-1 represents a high-risk model of immune–infection–cancer interaction, where patients develop oral and esophageal SCC decades earlier than the general population, in the absence of conventional carcinogens and with a predilection for Candida-colonized sites. The reproducible incidence of ~10% across independent cohorts, the balanced gender distribution and the absence of classical risk factors suggest an autoimmune-driven carcinogenic pathway. This makes APS-1 a valuable human model of “field cancerization”, which is not caused by external toxins but by the interplay of intrinsic immune defects and chronic infections. Despite these insights, several important limitations should be acknowledged. First, the available evidence is largely based on isolated case reports and small case series, which limits generalizability and precludes statistical analysis. Second, the absence of standardized diagnostic, therapeutic, and follow-up protocols introduces considerable heterogeneity. Third, a significant publication bias is likely present: severe, atypical, or outcome-rich cases are more likely to be reported, while milder or negative cases may remain unpublished. This may lead to an overestimation of cancer risk and mortality in APS-1 patients. These constraints underscore the need for prospective registries and collaborative multicenter efforts to capture the full clinical spectrum. Additional limitations stem from the high risk of bias inherent in the data sources. Due to the absence of control groups, it is not possible to estimate the relative risk of malignancy in APS-1 patients compared to unaffected individuals or APS-1 patients without cancer. Furthermore, key confounding factors, such as chronic Candida infection, duration and type of antifungal therapy, or the presence of modifying genetic variants, were not systematically assessed across cases. Treatment modalities (surgery, radiotherapy, chemotherapy) were reported, but outcomes were not stratified by modality or timing, limiting conclusions about therapeutic efficacy. The included cases predominantly originate from a limited number of countries, which may restrict generalizability to broader populations. Lastly, survival outcomes are derived from a small, heterogeneous cohort and should be interpreted with caution.

The consistent clinical pattern of early-onset squamous cell carcinoma (SCC) in APS-1 occurring at Candida-colonized sites without conventional carcinogenic exposure suggests that the underlying mechanism of carcinogenesis differs from that in sporadic oral cancers [2,40]. At the center of this process is a profound immunological defect caused by AIRE mutations that abrogate central tolerance and promote the formation of high affinity auto-antibodies. Among the most clinically relevant are neutralizing autoantibodies against IL-17A, IL-17F and IL-22, cytokines that play an indispensable role in mucosal immunity. The IL-17/IL-22 axis is responsible for controlling epithelial defense against Candida albicans by inducing antimicrobial peptides, recruiting neutrophils and maintaining the integrity of the epithelial barrier [3,41,42]. In APS-1, neutralization of these cytokines results in an inability to eradicate Candida, leading to chronic mucocutaneous candidiasis (CMC) in early childhood. This defect is exacerbated by near-universal neutralizing autoantibodies to type I interferons (IFN-α and IFN-ω), which play a central role in tumor immune surveillance of tumors by activating dendritic cells and cytotoxic T lymphocytes and promoting tumor antigen presentation. Taken together, these defects lead to a double susceptibility: on the one hand, the mucosa is chronically colonized by a pathogenic fungus and, on the other hand, the immune system is unable to efficiently recognize and eliminate premalignant or malignant clones [12,43].

Persistent colonization with Candida albicans acts as a direct carcinogenic factor via several molecular mechanisms. One important effector molecule is candidalysin, a pore-forming peptide toxin secreted by Candida during the hyphal phase. Candida lysin has a dual effect: it damages the epithelial membranes and triggers cellular stress responses, and it abnormally activates the epidermal growth factor receptor (EGFR). Activation of the MAPK signaling pathway (ERK1/2, p38), which is downstream of EGFR, leads to the induction of transcription factors such as c-Fos and c-Jun, which promote cell proliferation, differentiation and the secretion of pro-inflammatory cytokines. While acute EGFR activity is protective in mobilizing the immune response, in the context of APS-1—in which Candida infection is never eradicated—it leads to chronic, pathological stimulation of mitogenic signaling, resulting in a state of epithelial hyperproliferation that is highly favorable for oncogenic transformation [37,44,45,46]. In addition to the signaling effects, Candida albicans contribute to carcinogenesis through the production of mutagenic metabolites. The fungal alcohol dehydrogenase pathway converts ethanol and other substrates into acetaldehyde, a class I human carcinogen that forms DNA adducts, induces point mutations and impairs DNA repair pathways [37,47,48]. Candida is also capable of nitrosation reactions and produces N-nitrosamines, known carcinogens in the upper aerodigestive tract. These metabolites accumulate in saliva and mucosal niches and expose basal keratinocytes to a constant mutagenic insult [37,47,48,49]. Histological studies of Candida-associated leukoplakia show higher rates of epithelial dysplasia and malignant transformation compared to Candida-negative lesions, demonstrating a causal relationship between chronic fungal metabolism and neoplastic progression [50].

The microenvironment of chronic candidiasis is dominated by pro-inflammatory cytokines, including IL-1β, IL-6, TNF-α and IL-23, which activate transcriptional programs associated with oncogenesis. Activation of NF-κB and STAT3 in epithelial cells provides sustained proliferation, angiogenesis and resistance to apoptosis. Activated neutrophils and macrophages recruited by these signals release reactive oxygen and nitrogen species that cause DNA double-strand breaks and base modifications that accumulate over time. The combination of mitogenic signaling, genotoxic metabolites and oxidative stress leads to a continuous cycle of damage and regeneration that predisposes the mucosa to dysplasia and malignant transformation [45,51,52]. Equally important is the failure of immune surveillance in APS-1. Type I interferons normally induce interferon-stimulated genes that enhance tumor antigen recognition and support cytotoxic T-cell responses. Neutralization of IFN-α and IFN-ω in APS-1 impairs dendritic cell maturation and T cell cross-priming, thereby weakening the recognition of transformed keratinocytes. In addition, autoreactive T cells maintain chronic inflammation of the mucosa but are unable to effectively kill tumors [53,54]. Thus, APS-1 represents a “perfect storm” of carcinogenesis in which deficient mucosal immunity allows persistent fungal colonization, fungal virulence factors activate epithelial oncogenic signaling pathways and systemic immune deficiencies prevent the elimination of dysplastic cells.

**Figure 3:**
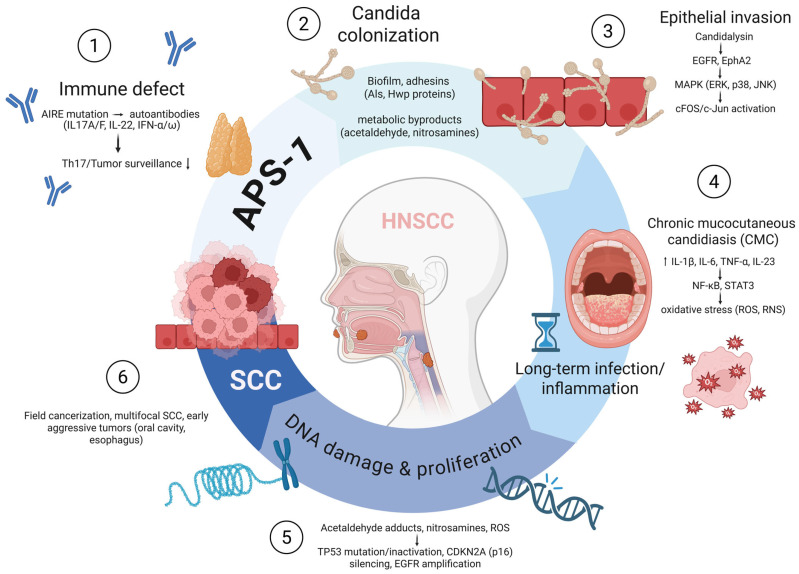
Molecular and clinical mechanisms linking APS-1 immune defects and chronic mucocutaneous candidiasis to squamous cell carcinoma (SCC).

The main immunological, microbial and molecular mechanisms that interact to drive carcinogenesis in APS-1 are summarized schematically in Figure 3.

APS-1 patients carry AIRE mutations that lead to autoantibodies against IL-17A/F, IL-22 and type I interferons (IFN-α/ω) and impair Th17 antifungal immunity and tumor surveillance. This defect allows persistent colonization with Candida albicans and the formation of biofilms with the production of carcinogenic by-products (acetaldehyde, nitrosamines). Invasion of the fungus by Candidalysin activates EGFR and EphA2 and triggers MAPK (ERK, p38, JNK) and c-Fos/c-Jun signaling. Chronic infection drives inflammatory cytokines (IL-1β, IL-6, TNF-α, IL-23) leading to NF-κB/STAT3 activation and oxidative stress (ROS, RNS). These processes culminate in DNA damage, TP53 inactivation, CDKN2A silencing, telomerase activation and EGFR amplification, leading to field cancerization and early multifocal SCC of the oral cavity and esophagus. Abbreviations: APS-1, autoimmune polyendocrine syndrome type 1; AIRE, autoimmune regulatory gene; IL, interleukin; IFN, interferon; CMC, chronic mucocutaneous candidiasis; EGFR, epidermal growth factor receptor; EphA2, ephrin type-A receptor 2; MAPK, mitogen-activated protein kinase; ROS, reactive oxygen species; RNS, reactive nitrogen species; TP53, tumor protein p53; CDKN2A, cyclin-dependent kinase inhibitor 2A (p16^INK4a^); SCC, squamous cell carcinoma. Created with www.app.biorender.com (accessed on 30 August 2025).

The concept that autoimmunity can act in a carcinogenic context is supported by data from several other autoimmune diseases. Large-scale cohort studies have shown that patients with psoriasis, systemic lupus erythematosus (SLE) and Sjögren’s syndrome have an increased risk of developing oral and other epithelial malignancies [55,56,57,58]. Psoriasis is a particularly illustrative example: it is triggered by excessive IL-17/IL-22 signaling and is characterized by hyperproliferation of keratinocytes. Molecules such as S100A7 (psoriasin) and squamous cell carcinoma antigen 2 (SCCA2), which are induced by IL-17/IL-22 in psoriasis, are also overexpressed in oral SCC and have been shown to promote keratinocyte proliferation and invasion [59,60,61]. This overlap suggests that both immunodeficiency (as in APS-1) and immune hyperactivation (as in psoriasis) may converge on common oncogenic pathways involving keratinocyte stress responses, cytokine looping and altered epithelial differentiation. In systemic lupus erythematosus and Sjögren’s syndrome, chronic immune activation and persistent tissue damage are similarly associated with a higher incidence of cancer, particularly lymphoma and mucosal SCC. This is a further precedent for autoimmunity as a driver of epithelial neoplasia [62,63,64]. Another important example is oral lichen planus (OLP), a chronic inflammatory autoimmune disease of the oral mucosa with a well-documented malignant potential. Although the reported risk varies, meta-analyses suggest that between 1 and 2% of OLP lesions undergo malignant transformation during long-term follow-up [65,66,67,68]. OLP shares with APS-1 the presence of chronic T cell-mediated epithelial inflammation, which induces oxidative stress, sustained release of cytokines and cycles of epithelial damage and regeneration that predispose to SCC. The inclusion of OLP in this context emphasizes that autoimmune-induced chronic inflammation, even in the absence of Candida or exogenous carcinogens, is sufficient to create a precancerous field in the oral cavity. Taking together, these conditions demonstrate that autoimmunity can predispose cancer through both immune deficiency and immune overactivation. Whether through a loss of cytokine-dependent defenses, as in APS-1, or through pathological overactivation of cytokine circuits, as in psoriasis and OLP, the end result is a convergence of similar molecular mechanisms that destabilize epithelial homeostasis and promote malignant transformation.

The increasing incidence of oral SCC at a young age in the general population is an important parallel to APS-1. Several population-based studies have reported an increase in oral cavity cancer in patients younger than 45 years, particularly tongue SCC in women, many of whom are non-smokers, non-drinkers and HPV-negative. In the United States, Surveillance, Epidemiology, and End Results (SEER) data show an annual increase of approximately 0.7% in men and 1.7% in women for tongue SCC in younger cohorts. Similar patterns have also been reported in Europe and Asia [69,70,71,72]. These “idiopathic” cancers of the oral cavity occurring in young people have a similar clinical phenotype to APS-1-associated malignancies, raising the possibility that at least a subset of these cases are due to a hidden immune disorder or chronic inflammation of the mucosa. Recent reviews have emphasized that clinicians should avoid dismissing young, risk factor-negative oral SCC as idiopathic without further investigation and should consider autoimmunity and immune dysregulation as plausible hidden causes [73,74].

The clinical implications of these mechanistic findings are profound. APS-1 patients should undergo lifelong oral and esophageal surveillance from adolescence, with careful examination of sites susceptible to candidiasis and a low threshold for biopsy of persistent lesions [2,75]. Aggressive treatment of candidiasis is essential but must always be weighed against the risk of fungal resistance. Long-term therapy with azoles (fluconazole, itraconazole) is effective in suppressing infection, but their use has been repeatedly associated with the emergence of azole-resistant Candida albicans and non-albicans species. Azole resistance is increasingly observed in the general population of patients with chronic mucocutaneous candidiasis. Re-resistant isolates of *C. albicans* are reported in up to 20–40% of long-term treated cases, and non-albicans species such as *C. glabrata* and *C. krusei*, which are inherently less sensitive to azoles, are frequently isolated in refractory infections [76,77]. These trends are clinically relevant for APS-1 because candidiasis is lifelong and exposure to antifungal agents is cumulative. Therefore, the selection pressure exerted by the chronic use of azoles in APS-1 patients is particularly strong, making the emergence of resistant Candida strains during long-term prophylaxis almost inevitable [77]. Rautemaa and colleagues reported that APS-1 patients receiving chronic azole prophylaxis frequently harbor azole-resistant Candida isolates, making eradication difficult and promoting persistent mucosal inflammation [22]. Alternative antifungal agents, including polyenes (topical amphotericin B, nystatin) or echinocandins, may attenuate resistance but are less suitable for long-term prophylaxis. Importantly, experimental models show that amphotericin B not only reduces fungal burden, but also attenuates epithelial DNA damage, downregulates COX-2 and PD-L1 expression and alters the tumor-promoting inflammatory microenvironment. These data suggest that antifungal agents can influence carcinogenesis at both microbiological and molecular levels [78,79]. Therefore, antifungal management—a strategic switch or combination of antifungal agents—should be considered in APS-1 to reduce resistance and ensure sustained infection control.

In addition to infection control, molecular profiling of APS-1-associated tumors should be a priority to identify therapeutic targets. Alterations such as the overexpression of EGFR, activation of STAT3, methylation of the p16 promoter and upregulation of interferon-stimulated genes such as IFI44L and ACSL4 have been identified in oral SCC and may also be relevant in APS-1-associated malignancies. This knowledge could pave the way for targeted therapies, including EGFR inhibitors, STAT3 blockade or immune checkpoint modulation, tailored to the particular immune landscape of APS-1 [80,81,82,83,84]. Another clinical implication is that young patients with oral SCC should be screened for autoimmune conditions such as APS-1, psoriasis or systemic lupus erythematosus, even in the absence of traditional risk factors. For oral and maxillofacial surgeons, pathologists and oncologists, the presence of oral SCC in a 30-year-old non-smoker should raise suspicion of an underlying immune-mediated disease, rather than being dismissed as idiopathic. In this way, APS-1 not only defines a rare monogenic syndrome but also serves as a human model for infection-induced field carcinogenesis, where the carcinogenic niche is characterized by immune failure and microbial persistence rather than exposure to exogenous carcinogens.

We acknowledge that our analysis is limited by the rarity of APS-1 and the nature of the available data. The 19 APS-1 cases included in our pooled analysis were predominantly from single case reports and small case series, which are inherently prone to publication bias and in which severe or unusual cases are likely to be over-represented. Many reports lacked detailed information, including detailed information on lifestyle, susceptibility to Candida strains, standardized follow-up and molecular tumor profiling. This variability inevitably limits the validity of the conclusions that can be drawn. A formal relative risk of oral cavity cancer in APS-1 could not be calculated as no controlled epidemiological studies are available. Nevertheless, consistent data from national cohorts in Finland and Italy clearly indicate that the risk is substantially increased, approaching 10% of patients in early to middle adulthood [9,17]. Another limitation is the heterogeneity of APS-1 itself: Some patients present with only the minimal triad of CMC, hypoparathyroidism and adrenal insufficiency, while others develop dozens of autoimmune complications. Our pooled cases likely exhibit a more severe phenotype, as these patients are more likely to have sought medical treatment for the development of cancer. It is plausible that APS-1 patients with residual AIRE function or milder immune dysregulation have a lower propensity to develop cancer, but this remains unclear. Additional factors such as Candida virulence—including azole resistance and candidalysin expression—nutritional status, oral hygiene and access to specialist care may influence outcomes but are not consistently reported. Although almost all tumors in APS-1 were squamous cell carcinomas, there are also reports of other malignancies (e.g., esophageal adenocarcinoma, gastric cancer and hematological disease). Our focus was limited to SCC of the head and neck, but clinicians should keep the broader spectrum of cancers with APS-1 in mind. In addition, the small number of reported APS-1 cases with malignancies, only 19 patients from published case reports and a supplementary cohort of 14, precludes any reliable estimation of incidence, risk stratification, or treatment outcomes. This limited sample size is an inherent constraint of the available literature. Finally, although the GRADE approach is a widely accepted framework for assessing the quality of evidence, its application was not feasible in this context due to the nature of the included studies, which were predominantly case reports and small series. We acknowledge this as a limitation of our scoping review. Furthermore, as this was a scoping review, we did not perform a formal critical appraisal of study quality or risk of bias, which inherently limits the strength of conclusions that can be drawn for clinical guidance. Despite these limitations, the convergence of findings from independent cohorts, national registries and case reports supports an association between APS-1, chronic candidiasis and early-onset oral SCC. Prospective, multicenter registries with systematic surveillance will be essential to determine actual cancer incidence, identify predictive biomarkers such as Candida load or autoantibody profiles and optimize prevention strategies.

## 4. Materials and Methods

This scoping review was conducted in accordance with the Preferred Reporting Items for Systematic Reviews and Meta-Analyses extension for Scoping Reviews (PRISMA-ScR) guidelines [19]. A completed PRISMA-ScR checklist for the abstract and manuscript is provided as Supplementary Material [85]. The review protocol, including the research question, eligibility criteria, and analytic approach, was defined as a priori to ensure transparency and reproducibility in the context of a rare condition with heterogeneous evidence. Although the protocol was not prospectively registered, all steps were undertaken in adherence to PRISMA-ScR recommendations.

A comprehensive literature search was carried out in PubMed/MEDLINE, Scopus, and the Web of Science Core Collection, covering all records from database inception through 1 July 2025. To maximize sensitivity, broad search terms were applied that captured both APS-1 and malignancies, thereby minimizing the risk of overlooking atypical or early reports. A detailed summary of the search strategy, including databases searched, keywords used, and the number of results retrieved from each source, is provided in Supplementary Table S1. The final search string combined synonyms for APS-1 (APS-1, APECED, “autoimmune polyendocrine syndrome type 1,” “autoimmune polyendocrinopathy candidiasis ectodermal dystrophy”) with general and specific malignancy terms (“cancer,” “carcinoma,” “squamous cell carcinoma,” “neoplasm,” “tumor,” “malignancy”). The search was restricted to English-language publications in humans, with no restrictions on year of publication. To ensure completeness, reference lists of all eligible articles and relevant reviews were screened manually.

Studies were considered eligible if they reported patients with a confirmed diagnosis of APS-1/APECED, established either by genetic confirmation of biallelic pathogenic variants in the AIRE gene or by fulfilling the accepted clinical diagnostic criteria, namely the presence of at least two of the three classical features of the syndrome: chronic mucocutaneous candidiasis, hypoparathyroidism, and primary adrenal insufficiency. Only cases with histologically confirmed malignant tumors of the head and neck region were included, encompassing oral cavity, tongue, lip, gingiva, palate, pharynx, larynx, esophagus, sinonasal tract, salivary glands, and skin of the head and neck. Exclusion criteria were malignancies outside the defined anatomical region, cases without a confirmed APS-1 diagnosis, animal studies, narrative reviews without original cases, conference abstracts without full text, and non-English publications. The inclusion criteria were guided by the PICOS framework, adapted to the context of a scoping review. The population of interest consisted of individuals diagnosed with autoimmune polyendocrine syndrome type 1 (APS-1/APECED). Since this was a non-interventional, observational review, there were no applicable interventions or comparators. The primary outcomes included the presence of head and neck malignancies, with available descriptive data on tumor type, location, treatment, and clinical course. Eligible study designs included case reports, case series, and retrospective cohorts that provided individual-level clinical and pathological information.

The screening process was undertaken independently by two reviewers. Titles and abstracts of all retrieved records were examined, and full texts of potentially eligible reports were assessed in duplicate. Any disagreements were resolved through consensus discussion. The entire selection process, including reasons for exclusion at the full-text stage, was documented in a PRISMA-ScR flow diagram (Figure 1).

Data extraction was also performed independently by two reviewers using a standardized template. Extracted variables included demographic details (age at cancer diagnosis, sex, country or region), features of APS-1 (mode of diagnosis, age of onset, components of the classical triad, presence and duration of CMC, other autoimmune manifestations), tumor characteristics (site, histology, grade, stage, multiplicity, recurrences, second primary tumors), exposure to conventional risk factors (tobacco, alcohol), treatment modalities (surgery, radiotherapy, chemoradiotherapy, or palliative measures), and outcomes (survival status, follow-up, recurrence). Whenever available, molecular and immunological data were also collected, such as AIRE genotype, autoantibody profiles, histopathological patterns, and expression of biomarkers.

Given the rarity of APS-1-associated head and neck malignancies and the heterogeneity of case reports, a descriptive synthesis was conducted. Continuous variables were summarized as medians and ranges, whereas categorical variables were reported as counts and percentages. Cases were stratified by tumor site and by the presence or absence of conventional risk factors. Where follow-up data were available, recurrence and mortality rates were calculated. Beyond descriptive analysis, findings were interpreted in the light of mechanistic hypotheses from experimental literature on candidiasis-associated carcinogenesis, particularly candidalysin-induced EGFR/MAPK activation, acetaldehyde and nitrosamine production, and chronic inflammation–driven mutagenesis.

In addition to case-level data, summary results from the Italian national APS-1 cohort [17] were incorporated to provide epidemiological context. This cohort reported 14 additional APS-1 patients with head and neck malignancies; however, because individual-level information was unavailable, these cases were described narratively and not integrated into the pooled analyses. Due to the descriptive nature of the available literature and the high degree of clinical heterogeneity, this review does not meet the methodological prerequisites for conducting a meta-analysis. No standardized outcome measures or comparator groups were available to support quantitative synthesis.

Although a formal risk of bias assessment is not mandated under PRISMA-ScR, we undertook a qualitative appraisal of the available literature. Particular attention was given to the completeness of case descriptions, the validity of APS-1 diagnosis, and the adequacy of tumor documentation. Given the predominance of case reports and small series, we employed a descriptive approach to risk of bias assessment, focusing on the presence or absence of key clinical and pathological details. Formal appraisal tools designed for comparative or interventional studies were not applicable due to the absence of control groups, consistent out-come measures, or quantitative data. We acknowledge that the evidence base is dominated by case reports and small case series, and therefore highly susceptible to publication bias, particularly with overrepresentation of severe cases.

## 5. Conclusions

Squamous cell carcinoma of the head and neck is a serious, life-threatening complication of APS-1 that typically occurs at a young age and without conventional carcinogenic exposure. The mechanisms observed in APS-1-associated SCC suggest a potentially distinct carcinogenic pathway compared to sporadic oral SCC, but further molecular validation is required. Despite the limitations of current evidence, the consistency of clinical findings in independent cohorts emphasizes a markedly increased risk of cancer in APS-1, estimated at 10% in early adulthood. While antifungal resistance is a relevant clinical concern, more evidence is needed to determine the optimal long-term antifungal strategies in APS-1 patients. APS-1 may represent a useful model to explore how autoimmunity and chronic infection could interact to promote epithelial carcinogenesis, though further mechanistic studies are needed to confirm this concept.

In light of these considerations, the clinical and biological insights derived from this scoping review should be interpreted cautiously. The findings are hypothesis-generating and point to important directions for future research, rather than providing definitive evidence for clinical practice or mechanistic conclusions.

## Figures and Tables

**Table 1:** Demographic, clinical, and genetic features of APS-1 patients with head and neck malignancies.

Case	Source	Age (y)	Sex	Country	APS-1 Onset (y)	APS-1 Components (Chronological)	AIRE Mutation	Autoantibodies	CMC Duration (y)
1	Richman et al., 1975 [20]	26	M	USA	22	HT(2), CMC(3), PA, PAI(22), GF(23), DM	NR	NR	23
2	Firth et al., 1997 [21]	21	F	Australia	16	HP(2), PA(8), PAI(16), POF, CMC	NR	NR	NR
3	Rautemaa et al., 2006 [22]	44	M	Finland	NR	A(18), AZ(27)	NR	NR	None
4	Rautemaa et al., 2006 [22]	33	M	Finland	NR	HP(9), PAI(9), K(10), CMC(14), PA(16)	NR	NR	19
5	Rautemaa et al., 2006 [22]	34	F	Finland	NR	CMC(0), HP(5), PAI(7), POF(27)	NR	NR	34
6	Rautemaa et al., 2006 [22]	29	F	Finland	NR	HP(1.9), PAI(5.4), A(10), POF(14.7), IN(19.3), CMC(13.4)	R257X/ R257X	NR	15.6
7	Rautemaa et al., 2006 [22]	40	M	Finland	NR	CMC(3.3), A(6), K(7), PAI(9.7), HP(16.4), DM(19.3)	NR	NR	36.7
8	Rautemaa et al., 2006 [22]	42	M	Finland	NR	HP(1.7), CMC(3.3.), K(9.7), V(11)	NR	NR	38.7
9	Böckle et al., 2010 [23]	40	F	Austria	40	HP(5), CMC(18), PAI(33), A(35), V(35)	c.967_979del13/ c.967_979del13	NR	22
10	Shephard et al., 2012 [24]	35	F	Australia	10	AS(0), HP(7), PAI(10), CMC	NR	NR	25+
11	Cormack et al., 2012. [25]	37	M	Ireland	27	HP(27), PAI(27), CMC(<29)	NR	NR	8+
12	Awad et al., 2015 [26]	34	F	Canada	0–6	CMC(0), ED(2), EH(2), K(2), HP(4), PAI(9), V(9), A(9), PA(18), HT(19), POF(19), DM(31)	NR	NR	34
13	Bruserud et al., 2016 [9]	35	M	Norway	4	HP(4), PAI(4), CMC(26), EH, A	c.967_979del13/ c.967_979del13	IFN-ω+, 21OH, AADC, IL-22, PCA, SCC, TGM4, TH	30
14	Bruserud et al., 2016 [9]	58	M	Norway	NR	HP, PAI(13), A	c.22C>T/ c.402delC	IFN-ω+, 21OH, 17OH, SCC, SOX10, TPH1	NR
15	Bruserud et al., 2016 [9]	35	M	Norway	12	HP(12), PAI(12), EH, B12(59)	c.967_979del13/ c.967_979del13	IFN-ω+, 17OH, IL-22, NALP5, SCC, TPH1	15+
16	Bruserud et al., 2018 [27]	45	F	Finland	1	HP(1), CMC(3), EH(6), HG(13), V(13), PAI(16), A(27), B12(38), M(30), DM1(31), AS(39), AT(47)	R257X/R257X	SCC, NALP5, IFN-ω	42
17	Bruserud et al., 2018 [27]	31	F	Finland	2	HP(2), PAI(5), EH(5), A(10), CMC(10), H(15), TN(19), AT(32)	R257X/R257X	17OH, SCC, NALP5, IL-22, IFN-ω	21
18	Bruserud et al., 2018 [27]	21	M	Norway	11	H(0), M(0), A, CMC(~3), PAI(11)	c.967_979del13/ c.967_979del13	21OH, 17OH, AADC, IL-22, SCC, TPH1, IFN-ω	15+
19	Bruserud et al., 2018 [27]	38	M	Israel	3	A(3), CMC, H, V, AS, HP (5), PAI	A374G/A374G	21OH, TPO	30+

AADC—aromatic L-amino acid decarboxylase; AIRE—Autoimmune Regulator; APS-1—autoimmune polyendocrine syndrome type 1; AS—asplenia; AT—autoimmune thyroiditis; AZ—azoospermia; B12—vitamin B12 deficiency; CMC—chronic mucocutaneous candidiasis; DM—diabetes mellitus; ED—enamel dysplasia; EH—enamel hypoplasia; GF—gonadal failure; H—hepatitis; HG—hypogonadism; HP—hypoparathyroidism; HT—hypothyroidism; IL-22—interleukin 22 autoantibodies; IFN-ω—interferon-omega; K—keratopathy; M—malabsorption; NALP5—NACHT leucine-rich-repeat protein 5; NR—not reported; PA—pernicious anemia; PAI—primary adrenal insufficiency (Addison’s disease); PCA—parietal cell autoantibodies; POF—premature ovarian failure; SOX10—SOX10 transcription factor; TGM4—transglutaminase 4 autoantibodies; IN/TN—(tubule)interstitial nephritis; TH—tyrosine hydroxylase; TPO—thyroid peroxidase autoantibodies; TPH1—tryptophan hydroxylase 1 autoantibodies; V—vitiligo; 17OH—17α-hydroxylase; 21OH—21-hydroxylase.

**Table 2:** Tumor localization, histopathological characteristics, treatment, and outcomes of APS-1 patients with head and neck malignancies.

Case	Tumor Site	Histology	Smoking/Alcohol	Treatment	Outcome	Follow-Up (mo)	Recurrence	SPT
1	lower lip, buccal mucosa, palate, gingiva	SCC Grade 1	NR	RT + CT	Died	8	Yes	Yes
2	buccal mucosa	SCC Grade 1, T4N0	No/Yes (minimal alcohol)	Primary RT; AF (ketoconazole, nystatin, amphotericin B)	Alive	60	No	No
3	buccal mucosa	SCC Grade 1, T2N1	Yes/Yes	Neoadjuvant RT; Surgery (IOE + ND)	Died	6.5	Yes (after 2.5 months)	No
4	buccal mucosa	SCC Grade 2, T1N2	Yes/NR	Palliative RT	Died	7	No	No
5	buccal mucosa	SCC Grade 1, T2N2	No/Yes (minimal alcohol)	Surgery (IOE + bilateral ND; AF (ketoconazole)	Died	24	Yes	No
6	lateral tongue	SCC Grade 1, T1N0	No/No	Surgery (IOE), AF (miconazole, fluconazole)	Alive	307	Yes (after 1 year)	No
7	esophagus	SCC Grade 1, T3N1	Yes/Yes	Surgery (esophagectomy), adjuvant CRT; AF (miconazole, flucoazole, ketoconazole)	Died	18	No	No
8	mandibular gingiva	SCC Grade 3, T4N1	Yes/Yes	CRT—refused radical surgery; AF (fluconazole)	Alive (terminal stage)	19	No	No
9	buccal mucosa, lower lip	SCC Grade 2, T2N0 SCC Grade 2, T1N0	No/No	Serial surgical excisions (laser resection & ablation); AF (amphotericin B, fluconazol)	Alive	12	Yes /after 2 months)	Yes
10	buccal mucosa	SCC Grade 1, T2N0	No/Yes (minimal alcohol)	Laser surgical excisions + ND; AF (amphotericin B, fluconazole, voriconazole)	Alive	48	No	Yes (lower lip, after 1 y; CIS- lip commissure, after 3 y)
11	esophagus	SCC Grade 2	NR	Neoadjuvant CRT; oesophagetomy	Alive	6	No	No
12	esophagus	SCC Grade 3	No/No	AF (long-term fluconazol, nystatin, micafungin); supportive care (advanced disease)	Died	5	No	No
13	tongue	SCC	NR	NR	Died	NR	No	
14	esophagus	AC Grade 3, T3N3M1	NR	NR	Died	NR	No	Yes (rectum)
15	lower lip	SCC	NR	NR	Alive	NR	Yes	
16	lateral tongue	SCC Grade 1, T1N0	Yes (~1–4 cigarettes/day) /Yes (minimal alcohol)	Surgery (IOE)	Alive	60	No	No
17	lateral tongue	SCC Grade 1, T1N0	No/Yes (~4 units/week)	Surgery (hemiglossectomy)	Alive	192	No	Yes/mandibular gingiva (PDT)
18	lateral tongue	SCC Grade 3, T3N1	No/No	Surgery (hemiglossectomy + ND), adjuvant CRT	Alive	7	No	No
19	lateral tongue	SCC T2N0	No/No	Surgery (partial glossectomy + SND); adjuvant RT; AF (nystatin, ketoconazol, fluconazole)	Died	27	Yes (after 2 years)	No

SCC—squamous cell carcinoma; AC—adenocarcinoma; CIS—carcinoma in situ; RT—radiotherapy; CT—chemotherapy; CRT—chemoradiotherapy; AF—antifungal therapy; IOE—intraoral excision; ND/SND—(selective) neck dissection; SPT—second primary tumor; NR—not reported.

## Supplementary Materials

The following supporting information can be downloaded at: https://www.mdpi.com/article/10.3390/ijms26188969/s1.

## Abbreviations

The following abbreviations are used in this manuscript:

APS-1	Autoimmune Polyendocrine Syndrome Type 1
APECED	Autoimmune Polyendocrinopathy–Candidiasis–Ectodermal Dystrophy
AIRE	Autoimmune Regulator
CMC	Chronic Mucocutaneous Candidiasis
SCC	Squamous Cell Carcinoma
HNSCC	Head and Neck Squamous Cell Carcinoma
IL	Interleukin
IFN	Interferon
EGFR	Epidermal Growth Factor Receptor
MAPK	Mitogen-Activated Protein Kinase
NF-κB	Nuclear Factor Kappa-light-chain-enhancer of activated B cells
STAT3	Signal Transducer and Activator of Transcription 3
TP53	Tumor Protein p53
CDKN2A	Cyclin Dependent Kinase Inhibitor 2A (p16^INK4a^)
PICOS	Population, Intervention, Comparator, Outcome, Study Design
PRISMA-ScR	Preferred Reporting Items for Systematic Reviews and Meta-Analyses Extension for Scoping Re-views
ROB	Risk of Bias
ROS	Reactive Oxygen Species
RNS	Reactive Nitrogen Species
OLP	Oral Lichen Planus
SEER	Surveillance, Epidemiology, and End Results Program
